# Gender Differences in Emotional Burden in Chronic Rhinosinusitis: The Role of Sleep-Related Impairment

**DOI:** 10.3390/jcm15103788

**Published:** 2026-05-14

**Authors:** Elena Cantone, Francesco Di Salle, Maria Raffaella Ambrosio, Cristiano Scandurra, Vincenzo Bochicchio

**Affiliations:** 1 Department of Pharmacy, Health and Nutrition Sciences, University of Calabria, 87036 Cosenza, Italy; elena.cantone@unical.it (E.C.); mariaraffaella.ambrosio@unical.it (M.R.A.); 2 Department of Medicine, Surgery and Dentistry, Scuola Medica Salernitana, Section of Neurosciences, University of Salerno, 84081 Salerno, Italy; fdisalle@unisa.it; 3 Department of Humanities, University of Naples Federico II, 80138 Naples, Italy; 4 Department of Humanities, University of Calabria, 87036 Cosenza, Italy; vincenzo.bochicchio@unical.it

**Keywords:** emotional distress, sex and gender differences, sleep disturbance, patient-reported outcomes, chronic rhinosinusitis

## Abstract

**Background/Objectives**: Chronic inflammatory conditions are increasingly conceptualized within biopsychosocial frameworks in which psychological processes shape illness experience and health-related quality of life (HRQoL). This study examined sex- and gender-related differences in emotional burden among patients with chronic rhinosinusitis with nasal polyps (CRSwNP), focusing on sleep disturbance as a potential factor associated with emotional distress. **Methods**: In a cross-sectional sample of 111 adults with CRSwNP, participants underwent clinical assessment and completed the Sino-Nasal Outcome Test. Independent-samples analyses examined sex differences in symptom severity and HRQoL domains. Hierarchical regression models tested whether emotional distress remained associated with sex after accounting for disease severity and sleep impairment. An exploratory mediation analysis with bootstrapped confidence intervals examined whether sleep disturbance accounted for this association. **Results**: Women reported greater emotional distress and sleep impairment than men despite comparable disease severity. Sex explained initial variance in emotional burden, while sleep impairment was the strongest predictor. Sex differences remained significant in the fully adjusted model. Mediation analyses suggested that sleep disturbance may be statistically associated with the relationship between sex and emotional distress. **Conclusions**: Emotional burden in CRSwNP reflects sex- and gender-related processes beyond clinical severity. Sleep disturbance may represent an important factor associated with emotional distress, supporting biopsychosocial models and integrated care.

## 1. Introduction

Chronic inflammatory conditions are increasingly conceptualized within a biopsychosocial framework in which biological disease processes interact with psychological factors, including emotional regulation, illness perception, and behavioral adaptation, to shape patient-reported outcomes and health-related quality of life [1,2]. Across chronic illnesses, emotional burden represents not only a consequence of symptom severity but also a dynamic process influenced by cognitive appraisal and self-regulatory mechanisms that shape adaptation to disease [3,4]. Sleep disturbances constitute an additional factor associated with both physiological dysregulation and emotional functioning, with bidirectional effects on mood and symptom perception demonstrated across clinical populations [5,6]. Understanding how these psychological processes interact with disease-specific factors is therefore central to contemporary health psychology research, particularly in conditions characterized by persistent symptoms and fluctuating disease trajectories.

Within this framework, sex- and gender-related differences have emerged as key determinants of illness experience and emotional adaptation. Evidence from health psychology suggests that women and men may differ in emotional processing, symptom perception, and help-seeking behaviors, partly reflecting biological influences, including sex-related differences in immune and hormonal functioning [7,8,9], as well as sociocultural norms governing emotional disclosure and health-related communication [10,11]. These differences may influence both subjective disease burden and the interpretation of patient-reported outcomes, highlighting the need for sex-informed analyses of psychological dimensions in chronic medical conditions [12].

Within this perspective, chronic medical conditions characterized by persistent symptoms, inflammatory processes, and substantial impacts on daily functioning represent particularly informative models for examining how biological and psychological factors interact to shape emotional burden and illness adaptation. As an illustrative model of chronic inflammatory disease with substantial psychological and functional burden, chronic rhinosinusitis with nasal polyps (CRSwNP) provides a valuable context for examining the interaction between biological processes and emotional adaptation. CRSwNP is a type 2-mediated inflammatory disorder of the sinonasal mucosa that significantly impairs health-related quality of life (HRQoL) and imposes a substantial burden on healthcare systems [13]. These observations support conceptualizing CRSwNP as a systemic inflammatory condition requiring comprehensive management [14]. Moreover, recent evidence highlights a bidirectional association between CRSwNP and mental health disorders, particularly anxiety and depression, underscoring the psychosomatic burden of the disease [15].

In this context, contemporary clinical practice increasingly incorporates validated Patient-Reported Outcome Measures (PROMs) to quantify disease burden and monitor treatment response. The 22-item Sino-Nasal Outcome Test (SNOT-22) is widely used to assess disease-specific HRQoL in patients with CRS [16,17]. Notably, the emotional subdomain of the SNOT-22 offers valuable insights into psychological dimensions of illness experience, reflecting growing recognition of the role of emotional and mental health factors in chronic rhinosinusitis and patient-reported outcomes research [15,18].

While traditional rhinologic research has largely emphasized physical and functional symptoms, emerging evidence highlights a high prevalence of psychological comorbidities in patients with CRS, including elevated rates of depression, anxiety, and emotional distress [19,20,21]. This relationship appears to be bidirectional, with chronic inflammation potentially contributing to affective disorders, and pre-existing psychological factors or vulnerabilities possibly intensifying symptom perception and disease burden [22]. Additionally, olfactory dysfunction—a hallmark of CRSwNP—has been shown to exert profound effects on mood and QoL, consistent with clinical and neurobiological evidence linking olfaction to emotional processing and behavior [23]. In this context, sleep disturbances may represent a key clinical factor associated with both physiological dysregulation and emotional burden, as research increasingly highlights the bidirectional relationship between sleep quality, emotional regulation, and psychological adjustment across clinical populations [5,6,24].

Within this framework, individual differences in emotional regulation and symptom appraisal may be further shaped by sex- and gender-related factors, which influence how emotional distress and sleep disturbances are experienced, interpreted, and reported in chronic illness. Emerging evidence suggests that men and women with CRS may differ not only in symptom profiles and treatment outcomes but also in the psychological interpretation and reporting of illness-related experiences [25]. However, systematic evaluations of sex- and gender-based differences across individual SNOT-22 domains remain limited, particularly with respect to emotional and sleep-related dimensions that may reflect distinct patterns of psychological adaptation. Investigating sex- and gender-specific symptom patterns and emotional responses in CRSwNP may therefore provide insights into how biological, psychological, and sociocultural factors interact to shape illness experience, ultimately informing more personalized and psychologically informed interventions.

The present study aimed to examine sex- and gender-related differences in health-related quality of life (HRQoL) among patients with CRSwNP, with a specific focus on psychological dimensions captured by the validated subdomains of the SNOT-22 (Nasal, Ear/Facial, Sleep, Function, Emotion). Based on prior evidence indicating sex differences in emotional regulation, symptom appraisal, and the reporting of psychological distress in chronic disease contexts [7], we hypothesized that women would report higher levels of emotional burden compared to men. Building on this initial hypothesis, the study further aimed to determine whether any observed sex differences in emotional burden would remain significant after accounting for objective indicators of disease severity. Specifically, we examined whether impairment in HRQoL and clinical severity indices would contribute to emotional distress and whether sex differences would persist after adjustment for these factors.

Grounded in a biopsychosocial and psychosomatic framework integrating inflammation, sleep regulation, and emotional processes, this study seeks to advance understanding of how gender-related factors shape psychological adaptation to chronic illness. By examining domain-specific patterns within patient-reported outcomes, the study aims to clarify the role of sleep-emotion interactions in the subjective experience of disease burden and to inform more nuanced, psychologically informed and patient-centered management strategies, including targeted interventions addressing both emotional well-being and sleep disturbances.

## 2. Materials and Methods

### 2.1. Procedures

A cross-sectional observational study was conducted to examine sex- and gender-related differences in psychological and health-related quality-of-life (HRQoL) outcomes among patients with chronic rhinosinusitis with nasal polyps (CRSwNP). The primary outcome consisted of comparing SNOT-22 subdomain scores based on the five-domain structure proposed by Khan et al. [26]. Data were extracted from outpatient medical records at the Otorhinolaryngology Unit of the University of Naples Federico II between January 2024 and January 2025.

The study was conducted in accordance with institutional ethical standards and the Declaration of Helsinki and adhered to STROBE guidelines for observational research. Ethical approval was obtained from the local Ethics Committee of the University of Naples Federico II (protocol no. 424/21, approved on 27 December 2021), and all participants provided written informed consent.

### 2.2. Participants

Inclusion criteria were: (1) age ≥ 18 years; (2) diagnosis of CRSwNP according to the European Position Paper on Rhinosinusitis and Nasal Polyps [13]; (3) biologic- and surgery-naïve status; and (4) receipt of standard medical therapy for ≥3 months prior to inclusion.

Exclusion criteria included pregnancy, immunosuppressive treatment, radiotherapy or chemotherapy within the current or previous five years, ongoing or recent systemic steroid therapy, diagnosed neurological or psychiatric disorders, history or clinical suspicion of eosinophilic granulomatosis with polyangiitis (EGPA), or evidence of non–type 2 inflammation-driven nasal polyposis.

### 2.3. Measures

Demographic variables included age, sex (male, female, or intersex), and gender identity (cisgender vs. transgender). Objective disease severity was assessed using nasal endoscopy scored with the Nasal Polyp Score (NPS) and computed tomography evaluated with the Lund–Mackay (LM) score. Sex was recorded as male/female from clinical records. Gender identity was assessed with a single item asking whether participants identified with their sex assigned at birth (cisgender vs. transgender); all participants were cisgender. Accordingly, references to “gender” in this manuscript refer to sociocultural correlates interpreted at the analytic level rather than measured gender-role constructs, in line with the Sex and Gender Equity in Research (SAGER) guidelines [27].

Symptom severity was assessed using Visual Analog Scales (VAS) evaluating nasal obstruction (VASn), rhinorrhea (VASr), olfactory dysfunction (VASo), pain (VASp), and sleep quality (VASsq), each ranging from 0 (no symptoms) to 10 (worst severity). The use of VAS for symptom assessment in CRS is recommended in the European Position Paper on Rhinosinusitis and Nasal Polyps [13].

HRQoL in chronic rhinosinusitis was assessed through the Sino-Nasal Outcome Test (SNOT-22) [26], a validated multidimensional patient-reported outcome measure. The instrument captures both physical symptom severity and psychosocial aspects of illness experience, including symptoms such as need to blow the nose and post-nasal discharge, as well as emotional responses such as sad and frustrated/restless/irritable. Subdomain scores were calculated according to the validated five-domain model, comprising nasal symptoms (SNOT22n), ear/facial symptoms (SNOT22ef), sleep dysfunction (SNOT22s), functional impairment (SNOT22f), and emotional distress (SNOT22e). Higher scores indicate greater symptom burden and reduced HRQoL. The alpha coefficient for the current sample was 0.86 for SNOT22n, 0.84 for SNOT22ef, 0.89 for SNOT22s, 0.92 for SNOT22f, 0.90 for SNOT22e, and 0.94 for the total scale.

Olfactory function was assessed using the Sniffin’ Sticks Identification Test (Burghart Messtechnik GmbH, Wedel, Germany; [28]). Odorants were presented in felt-tip pens, and participants identified 16 common odors using a multiple-choice format, selecting the correct option from four descriptors. Total scores ranged from 0 to 16, with higher scores indicating better olfactory identification.

### 2.4. Statistical Analysis

Statistical analyses were conducted using IBM SPSS Statistics version 27, with the level of statistical significance set at *p* < 0.05. Descriptive statistics (means and standard deviations) were calculated for all demographic and clinical variables. Independent-samples *t* tests were performed to explore potential sex differences across the main study variables. Effect sizes for independent-samples *t* tests were calculated using Cohen’s *d*, with values of 0.20, 0.50, and 0.80 interpreted as small, medium, and large effects, respectively [29].

To further investigate sex differences and to determine whether any observed differences in the outcome variable remained significant after accounting for relevant clinical factors, hierarchical multiple linear regression analyses were conducted. Sex was entered in the first step of the model, followed by additional clinically relevant variables entered in subsequent steps according to theoretical and clinical rationale. This approach allowed us to examine the incremental contribution of these variables and to verify whether sex remained independently associated with the outcome after adjustment.

Effect sizes were estimated using Cohen’s *f*^2^, with values of 0.02, 0.15, and 0.35 indicating small, medium, and large effects, respectively [29]. Assumptions of linear regression were assessed by inspecting variance inflation factors (VIFs), residual plots, and standardized residuals. VIF values below 5 were considered acceptable, and no substantial violations of linearity, homoscedasticity, or normality of residuals were observed.

Following the regression analyses, an exploratory mediation analysis was conducted to further examine whether sleep disturbance was statistically associated with the relationship between sex and emotional distress. The mediation model was estimated using the PROCESS macro version 5.0 for SPSS (Model 4) with 5000 bootstrap samples. Bias-corrected 95% confidence intervals were used to determine the significance of indirect effects.

## 3. Results

**Figure 1:**
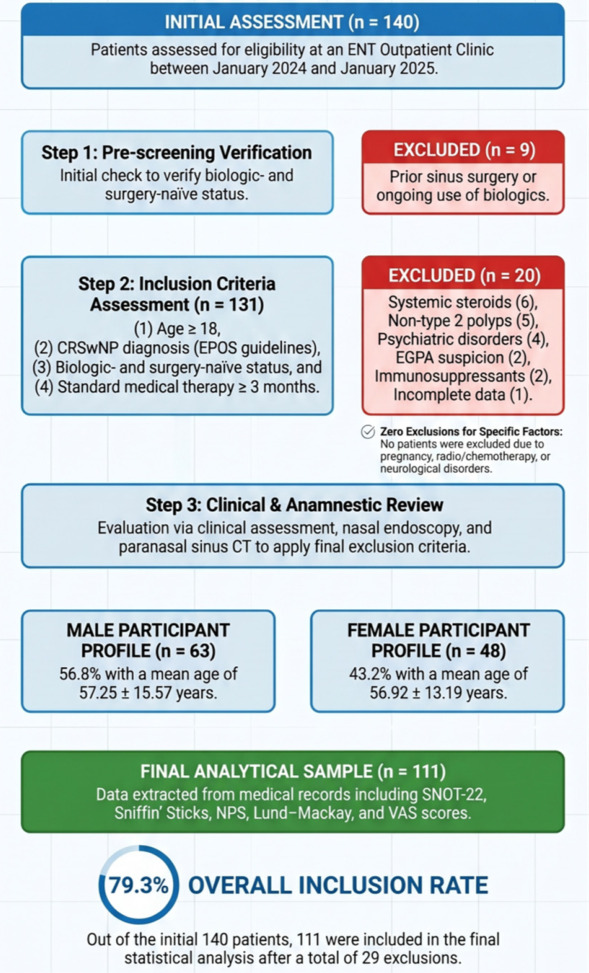
Flow Chart Illustrating the Patient Selection Process.

A total of 140 patients were initially assessed for eligibility between January 2024 and January 2025. Of these, 29 were excluded during the screening process, including 9 at pre-screening and 20 after assessment of inclusion and exclusion criteria. The final analytical sample comprised 111 participants (63 males and 48 females). The patient selection process is illustrated in Figure 1.

### 3.1. Sex Differences in Demographic and Clinical Variables

Descriptive statistics and sex comparisons are presented in Table 1. The sample had a mean age of 57.11 years (SD = 14.53). No significant sex differences were found in age. Females reported significantly higher NPS scores than males, with a medium effect size (*d* = 0.61), indicating greater disease severity. No significant sex differences emerged in SS scores or Lund–Mackay (LM) scores.

Regarding VAS measures, females showed higher mean scores across all domains; however, statistical significance was reached only for VAS sleep, with a small-to-medium effect size (*d* = 0.38). Similarly, females reported higher scores on all SNOT-22 subscales, with a significant difference observed in the SNOT-22 Emotion domain. Because the sample included women across a wide age range, an additional exploratory comparison was conducted within the female subsample between women of reproductive age and postmenopausal women. Twenty-three women were postmenopausal, and no significant differences were found between these groups in the Emotion subdomain (*p* = 0.30).

Total SNOT-22 scores were significantly higher in females compared to males (56.85 ± 24.91 vs. 47.24 ± 21.21, *p* < 0.05), with a small-to-medium effect size (*d* = 0.42). In addition, females reported greater emotional distress (SNOT-22e: 7.85 ± 4.95 vs. 5.16 ± 4.17, *p* < 0.01), with a medium effect size (*d* = 0.59), and poorer sleep quality (VAS sleep: 5.14 ± 2.83 vs. 3.99 ± 3.21, *p* < 0.05), with a small-to-medium effect size (*d* = 0.38). Overall, these findings indicate that females experienced greater symptom burden, particularly in emotional and sleep-related domains.

### 3.2. Associations of Gender, Disease Severity, and Sleep Impairment with Emotional Distress

To clarify the relative contribution of demographic and clinical factors to emotional distress, and given the observed sex differences in sleep and emotional symptoms, we conducted a hierarchical multiple linear regression analysis with emotional distress as the outcome and gender, objective disease severity (NPS), and sleep impairment as predictors. Given the emergence of significant sex differences—particularly in emotional symptoms and sleep impairment—and considering that NPS reflects objective disease severity, the analysis aimed to determine whether gender differences in emotional distress remained significant after accounting for clinical severity and sleep-related symptoms.

A sensitivity power analysis was conducted using G*Power 3.1 (F tests, linear multiple regression: fixed model, *R*^2^ deviation from zero). With *N* = 111, *α* = 0.05, and three predictors, the study had 80% power to detect effects of at least *f*^2^ = 0.10, corresponding to a small-to-medium effect according to Cohen’s [29] conventions. These results indicate that the sample size provided adequate statistical power to detect effects of practical relevance.

Hierarchical multiple linear regression results are presented in Table 2. All VIFs were within acceptable limits (from 0.904 to 1.106), indicating no multicollinearity concerns. Inspection of residual diagnostics further indicated no substantial violations of linearity, homoscedasticity, or normality assumptions.

In Step 1, sex was entered into the model and explained 9% of the variance in emotional distress (*R*^2^ = 0.09), with a small-to-medium effect size (*f*^2^ = 0.09). Specifically, being female was associated with higher levels of emotional distress.

In Step 2, objective disease severity (NPS) was added to the model, explaining a non-significant additional 0.3% of the variance (Δ*R*^2^ = 0.003). Disease severity was not significantly associated with emotional distress and did not meaningfully increase the explained variance. Importantly, sex remained a statistically significant predictor after adjustment for objective disease severity, suggesting that the association between sex and emotional distress was independent of nasal polyposis severity.

In Step 3, sleep impairment was included in the model and accounted for a significant incremental 22% of the variance in emotional distress (Δ*R*^2^ = 0.22). Sleep impairment was positively associated with emotional distress, such that greater sleep impairment corresponded to higher levels of emotional distress. Although sleep impairment emerged as the strongest predictor in the model, gender continued to be significantly associated with emotional distress in the fully adjusted model.

The final model explained 31% of the variance in emotional distress (*R*^2^ = 0.31), corresponding to a medium-to-large effect size (*f*^2^ = 0.45). Overall, these findings indicate that sex differences in emotional distress persist even after accounting for both disease severity and sleep impairment, with sleep problems representing the most powerful predictor but not fully accounting for the observed gender effect.

Notably, the observed effect size for the final model (*f*^2^ = 0.45) substantially exceeded the minimum detectable effect identified in the sensitivity analysis (*f*^2^ = 0.10), further supporting the adequacy of the sample size and the robustness of the findings. Given that sleep impairment emerged as the strongest predictor of emotional distress while sex differences remained significant in the fully adjusted model, an additional mediation analysis was conducted to explore whether sleep impairment might partially account for the observed association between sex and emotional distress.

### 3.3. Additional Mediation Analysis

The mediation analysis was performed using the PROCESS macro for SPSS (Model 4; 5000 bootstrap samples), with emotional distress as the outcome variable, sex as the predictor, and sleep disturbance as the statistical intervening variable. Consistent with the regression models, objective disease severity (NPS) was included as a covariate.

The total effect of sex on emotional distress was significant (*B* = 2.70, *p* = 0.002), indicating that women reported higher levels of emotional distress compared to men. Sex was positively but only marginally associated with sleep disturbance (*B* = 1.15, *p* = 0.052), suggesting a tendency for female participants to report greater sleep impairment.

Sleep disturbance was associated with emotional distress, and this association differed by sex, with a stronger effect observed among women than among men. The indirect effect of sex on emotional distress through sleep disturbance was significant (*B* = 1.19, *BootSE* = 0.59, 95% *CI* [0.06, 2.39]), indicating that sleep disturbance was statistically associated with the relationship between sex and emotional distress.

**Figure 2:**
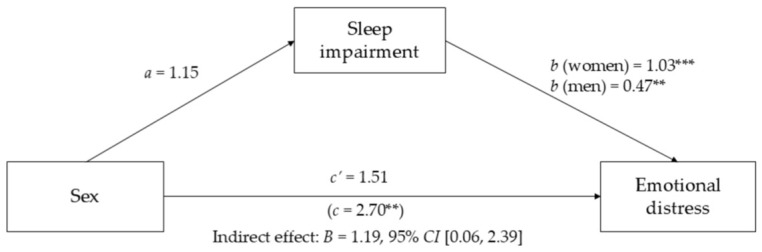
Mediation Model Illustrating the Association Between Sex, Sleep Impairment, and Emotional Distress. Notes: Unstandardized coefficients are reported. Objective disease severity was included as a covariate. ** *p* < 0.01, *** *p* < 0.001.

After inclusion of sleep disturbance in the model, the direct effect of sex on emotional distress was reduced and became non-significant (*B* = 1.51, *p* = 0.061), suggesting that sleep disturbance may be statistically involved in the association between sex and emotional distress. As in the regression models, objective disease severity did not significantly contribute to the model. The mediation model is illustrated in Figure 2.

## 4. Discussion

**Figure 3:**
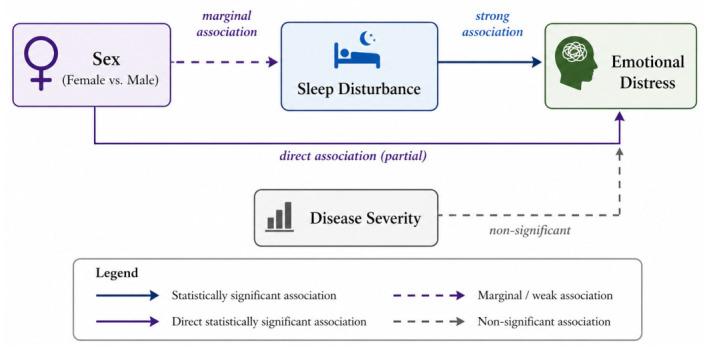
Conceptual Model of Sex Differences in Emotional Distress in Chronic Rhinosinusitis with Nasal Polyps (CRSwNP). Notes: Conceptual model summarizing the associations among sex, sleep disturbance, and emotional distress in patients with CRSwNP. Solid lines indicate statistically significant associations, whereas dashed lines represent non-significant or marginal associations. Sleep disturbance showed the strongest association with emotional distress, while sex remained directly associated with emotional distress after adjustment. Disease severity was included as a covariate but was not significantly associated with emotional distress.

The current study examined sex-related patterns of psychological burden in CRSwNP, focusing on domain-specific patient-reported outcomes and on the role of sleep–emotion coupling in emotional distress. The findings indicate that sex differences were primarily concentrated in the emotional domain of HRQoL and remained significant after accounting for objective disease severity and sleep-related impairment. Sleep impairment emerged as the strongest predictor of emotional distress, substantially increasing the explained variance, although it did not fully account for the observed sex differences. Overall, these findings suggest that sleep-related processes may partly account for sex disparities in emotional burden, as also reflected in the conceptual model (Figure 3).

Consistent with psychosomatic research, sex-related biological differences—including hormonal and immune mechanisms—may contribute to differential vulnerability to chronic inflammatory conditions and associated psychological outcomes [12,30]. While overall SNOT-22 scores were higher in women, statistically significant sex differences emerged only in the “Emotion” subdomain, with a medium effect size, despite comparable objective disease severity. Notably, the correlation between sleep and emotional symptoms was substantially stronger in women, suggesting that sleep disruption may disproportionately contribute to emotional burden in female patients.

Our findings are consistent with prior CRS research showing that women report greater subjective symptom burden on PROMs despite comparable objective disease indices (e.g., Lund–Mackay CT scores, NPS) [19,31]. Retrospective CRSwNP cohorts have similarly shown higher total SNOT-22 scores among women even with comparable imaging results [19]. However, relatively few studies have examined differences at the subdomain level, and disease-specific analyses (e.g., in Aspirin-Exacerbated Respiratory Disease) have yielded minimal or non-significant sex differences in this regard [32]. By identifying a specific discrepancy within the “Emotion” domain, our results refine current understanding of sex disparities in CRSwNP and align with broader evidence indicating higher emotional distress among women with chronic conditions [7,22,31]. In addition, evidence from olfactory dysfunction research suggests that olfaction-related HRQoL is closely linked to psychological distress, with women reporting greater impairment in response to chemosensory deficits [33]. Together, these findings support a framework in which sensory and subjective symptom processes are associated with greater emotional burden in women. In addition, rhinosinusitis should be considered within a broader network of overlapping clinical phenotypes, including migraine and sinus headache, which are often difficult to distinguish due to shared symptom profiles and partially overlapping pathophysiological mechanisms. These conditions are frequently co-reported and may reflect fluid and interacting phenotypes rather than clearly separable disorders, with hypersensitivity and inflammatory processes contributing to their co-occurrence [34]. This overlap may further contribute to variability in symptom perception and reporting, potentially amplifying the subjective burden experienced by patients, particularly in women.

An additional aspect that warrants consideration concerns the distinction between pain directly attributable to sinonasal inflammation and pain reflecting migraine-like processes that may be elicited or exacerbated in the context of CRSwNP. Although pain-related symptoms were included in the overall symptom assessment (e.g., VAS pain), the present study did not specifically differentiate between sinusitis-related pain and migraine-like headache. This distinction is clinically relevant, as a substantial proportion of patients reporting “sinus headache” may in fact present with migraine or overlapping headache phenotypes. From this perspective, part of the emotional burden observed in our sample may reflect not only inflammatory disease processes but also the contribution of headache-related mechanisms with distinct neurobiological underpinnings.

A multi-level explanatory framework best accounts for these sex differences. Biologically, sex-based disparities in immune and neuroimmune signaling—modulated by sex hormones and sex chromosome effects—can influence symptom perception and mood-related outcomes in the context of chronic inflammation [8,9]. Among the mechanisms potentially underlying these differences, sleep-emotion interactions emerge as a particularly salient factor. Sleep disturbances may represent an important component associated with both physiological dysregulation and emotional burden, as women show a higher prevalence of insomnia and report poorer subjective sleep quality across the lifespan, with increased vulnerability during the menopausal transition [6,35,36]. In line with evidence from the sleep-disordered breathing literature, women often report greater subjective symptom burden and psychological distress despite comparable or even milder objective disease severity, and frequently present with less typical symptoms such as insomnia, fatigue, and mood disturbances, which may contribute to under-recognition and under-diagnosis in clinical settings [37]. These patterns have been partly attributed to hormonal modulation, particularly the role of estrogen in sleep regulation and its decline during menopause, further supporting a biological basis for increased sleep vulnerability in women.

However, the exploratory comparison between premenopausal and postmenopausal women in the present study should be interpreted with caution, as the lack of statistically significant differences may reflect limited statistical power due to the small subsample size rather than the absence of a true effect. Prospective and meta-analytic studies further demonstrate that sleep disturbance is a robust predictor of subsequent emotional dysregulation and affective disorders [5,24,38,39]. These findings support the hypothesis that sleep disruption may exacerbate emotional symptoms, especially in women, a pattern corroborated by our data showing stronger sleep-emotion coupling in female participants. This aligns with contemporary neuroimmune models linking chronic inflammation to mood and affective symptoms [40,41].

In line with this interpretation, the exploratory mediation analysis provided additional support for the statistical involvement of sleep-related processes in the association between sex and emotional burden. Specifically, sleep disturbance appeared to be statistically associated with the relationship between sex and emotional distress, suggesting that sleep impairment may contribute to the observed pattern of associations, particularly among female patients. At the same time, this finding should not be interpreted as evidence of a causal mechanism, given the cross-sectional design of the study. Rather, sleep-related impairment may be understood as one component within a broader biopsychosocial network in which biological susceptibility, psychological coping styles, and sociocultural patterns of emotional expression interact to shape patients’ subjective experience of illness.

From a psychological perspective, women tend to report higher levels of repetitive negative thinking, particularly rumination, which is associated with increased risk for internalizing symptoms such as anxiety and depression [42,43]. In terms of emotion regulation, cognitive reappraisal appears beneficial across sexes, with evidence suggesting that women tend to ruminate more while men are more likely to suppress emotional expression [44]. Neuroimaging studies further support sex-based differences in neural circuitry involved in emotion regulation, providing a potential neuropsychological basis for the greater emotional burden reported by women with CRSwNP [45]. These findings may also explain the lower “Emotion” subdomain scores reported by men, consistent with higher suppression and reduced affective disclosure.

Sociocultural factors may further contribute to these patterns. Gendered norms surrounding emotional expression encourage greater emotional disclosure in women and greater suppression of distress in men, potentially influencing symptom awareness, reporting, and help-seeking behaviors [10,11,46]. Recent studies demonstrate that these disclosure patterns are context-sensitive and gender-contingent, reinforcing the notion that emotional symptom reporting is influenced by sociocultural expectations [47]. Accordingly, men may under-report emotional symptoms on PROMs, while women’s greater expressiveness may amplify measured burden beyond what is attributable to biological or psychological factors alone. Collectively, these biological, psychological, and sociocultural processes provide a comprehensive framework for understanding sex-related differences in SNOT-22 “Emotion” scores. They help explain both the heightened self-reported emotional burden in women and the comparatively lower scores in men, even after adjustment for objective disease severity and sleep impairment. Taken together, these findings support a biopsychosocial conceptualization of chronic inflammatory disease in which gendered patterns of psychological adaptation—particularly sleep-emotion interactions—shape the subjective experience of illness beyond objective disease severity.

With regard to the clinical implications, these findings underscore the importance of an integrated biopsychosocial approach to care. Clinicians should incorporate routine screening for sleep disturbances and emotional distress using brief, validated instruments, and longitudinally monitor SNOT-22 subdomain trajectories to identify clinically meaningful changes. When impairment is detected, targeted interventions—such as brief psychological support, structured sleep hygiene education, and stress-management programs—should be made available within a stepped-care framework.

Importantly, providers must account for sex-related differences in emotional disclosure. Men may under-report affective symptoms due to a greater tendency toward suppression and reduced help-seeking. Clinicians should therefore use proactive, normalizing language, seek collateral information when appropriate, and repeat PROM assessments to mitigate the risk of under-recognizing emotional burden.

These findings also support the adoption of sex-sensitive approaches to CRSwNP management, in which assessment and intervention strategies are tailored to account for differences in emotional expression, symptom perception, and sleep-related impairment between male and female patients. This highlights the importance of a differential diagnostic approach that accounts for overlapping, gender-sensitive, and sleep-related phenotypes in patients with CRSwNP.

### 4.1. Limitations

First, the cross-sectional design precludes causal inference and limits conclusions regarding the temporal ordering of sleep and emotional processes. Accordingly, the mediation analysis should be interpreted as exploratory and hypothesis-generating.

Second, the exclusion of patients with formal psychiatric diagnoses may not fully eliminate subclinical symptoms or ensure broader external validity.

Third, sleep disturbance was assessed using the SNOT-22 sleep subdomain, which captures subjective, disease-specific sleep impairment rather than objective sleep parameters. As such, sex differences in self-reported sleep disturbance may partly reflect differences in symptom perception or reporting styles, with women potentially reporting greater perceived impairment. This also raises the possibility of shared method variance, which may have contributed to the observed association between sleep disturbance and emotional distress.

Finally, sleep disturbance should be interpreted as one factor associated with emotional distress rather than a causal mechanism.

### 4.2. Future Research Directions

Future research should employ longitudinal designs to clarify the temporal relationships between sleep disturbance and emotional distress, and should incorporate objective sleep measures (e.g., actigraphy or polysomnography) alongside patient-reported outcomes. In addition, further studies are needed to explore sex-specific pathways linking inflammatory processes, sleep regulation, and psychological functioning, potentially integrating biological markers and neuroimmune indicators.

Future investigations should also examine whether interventions targeting sleep disturbance (e.g., behavioral sleep interventions or integrated care approaches) may differentially impact emotional outcomes across sexes. The inclusion of more comprehensive psychological assessments (e.g., measures of emotion regulation, rumination, and coping strategies) would further contribute to a more precise characterization of the mechanisms underlying sex-related differences in emotional burden.

Larger and more diverse samples would allow more robust examination of sex- and gender-related differences across clinical subgroups, as well as the role of contextual and sociocultural factors in shaping symptom perception and reporting. As biologic therapies gain prominence in CRSwNP management, upcoming clinical trials and real-world studies should pre-specify sex-disaggregated endpoints and examine whether improvements in SNOT-22 Sleep and Emotion domains parallel changes in type 2 inflammatory activity.

Finally, future research should incorporate more refined phenotyping of pain and headache characteristics in order to disentangle overlapping clinical presentations—such as sinusitis-related pain and migraine-like headache—and to better clarify their respective contributions to psychological distress.

## 5. Conclusions

The present findings highlight the importance of psychological dimensions in chronic inflammatory disease, showing that emotional burden represents a sex- and gender-differentiated component of illness experience. Female patients reported greater emotional distress, and these differences remained significant even after accounting for objective disease severity and sleep-related impairment. Hierarchical analyses indicated that sleep impairment was the strongest predictor of emotional distress, substantially increasing the explained variance, although it did not fully account for the observed sex differences. Complementary mediation analyses suggested that sleep disturbance may represent one factor associated with the observed relationship between sex differences and emotional distress, consistent with a biopsychosocial framework in which biological, psychological, and sociocultural factors jointly shape emotional adaptation to chronic illness. Clinically, these findings underscore the importance of sex- and gender-informed assessment of sleep and emotional health, as well as psychologically integrated approaches within multidisciplinary care models.

## Figures and Tables

**Table 1:** Descriptive Statistics of Demographic and Clinical Variables for the Total Sample and by Sex.

	Total Sample (*n* = 111)	Male (*n* = 63)	Female (*n* = 48)			
	M ± SD	M ± SD	M ± SD	*t*	*p*	*d*
Age	57.11 ± 14.53	57.25 ± 15.57	56.92 ± 13.19	0.12	0.904	0.02
NPS	6.22 ± 1.11	5.94 ± 1.1	6.6 ± 1.06	−2.78 **	0.007	0.61
SS	7.08 ± 3.80	7.06 ± 3.79	7.11 ± 3.87	−0.06	0.953	0.01
LM	19.42 ± 2.24	19.07 ± 2.16	19.90 ± 2.38	−0.89	0.384	0.37
VASn	7.02 ± 2.58	6.75 ± 2.94	7.4 ± 1.98	−1.29	0.179	0.25
VASr	5.63 ± 3.06	5.49 ± 3.11	5.83 ± 3.01	−0.57	0.571	0.11
VASo	6.90 ± 2.95	6.51 ± 3.20	7.41 ± 2.53	−1.61	0.100	0.31
VASp	3.18 ± 2.88	2.78 ± 2.65	3.71 ± 3.09	−1.71	0.090	0.32
VASsq	4.49 ± 3.09	3.99 ± 3.21	5.14 ± 2.83	−1.97 *	0.048	0.38
SNOT22	51.40 ± 23.28	47.24 ± 21.21	56.85 ± 24.91	−2.19 *	0.030	0.42
SNOT22n	22.64 ± 9.04	21.30 ± 9.23	24.40 ± 8.57	−1.80	0.074	0.35
SNOT22ef	7.04 ± 4.63	6.43 ± 4.38	7.83 ± 4.88	−1.59	0.114	0.30
SNOT22s	8.64 ± 5.91	8.10 ± 5.68	9.35 ± 6.20	−1.11	0.268	0.21
SNOT22f	6.66 ± 4.67	6.17 ± 4.21	7.29 ± 5.19	−1.25	0.227	0.24
SNOT22e	6.32 ± 4.70	5.16 ± 4.17	7.85 ± 4.95	−3.11 **	0.002	0.59

Notes: n = number; M = Mean; SD = Standard Deviation; NPS = Nasal Polyp Score; SS = Sniffing Sticks; LM = Lund-Mackay Score; VASn = nasal obstruction; VASr = rhinorrhea; VASo = olfactory dysfunction; VASp = pain; VASsq = sleep quality; SNOT22 = Sino-Nasal Outcome Test; SNOT22n = nasal (rhinologic) symptoms; SNOT22ef = ear/facial symptoms; SNOT22s = sleep dysfunction; SNOT22f = functional impairment; SNOT22e = emotional distress; *t* = t-value; *d* = Cohen’s d. ** *p* < 0.01; * *p* < 0.05.

**Table 2:** Hierarchical Multiple Linear Regression of Emotional Distress on Gender, Disease Severity, and Sleep Impairment.

	Emotional Distress		
	*B* (*SE*)	*β*	95% *CI*
*Step 1—Gender*			
Gender (male)	3.20 (1.02)	0.33 **	1.17, 5.24
	*R*^2^ = 0.09; *F* = 9.79 **		
*Step 2—Disease severity*			
Gender (male)	3.03 (1.08)	0.31**	0.89, 5.17
Disease severity	0.26 (0.48)	0.06	−0.67, 1.22
	*R*^2^ = 0.09; Δ*R*^2^ = 0.003; *F* = 5.00 **		
*Step 3—Sleep Impairment*			
Gender (male)	2.59 (0.94)	0.27 **	0.71, 4.46
Disease severity	0.13 (0.42)	0.03	−0.71, 0.97
Sleep impairment	0.75 (0.15)	0.47 ***	0.46, 1.04
	*R*^2^ = 0.31; Δ*R*^2^ = 0.22 ***; *F* = 13.07 ***		

Notes. *B* = Unstandardized regression coefficient; *SE* = Standard error; *CI* = Confidence interval; *β* = Standardized regression coefficient; *R*^2^ = R-square; Δ*R*^2^ = Change in R^2^. *** *p* < 0.001; ** *p* < 0.01.

## Data Availability

Data are available from the corresponding author upon reasonable request.

## Abbreviations

The following abbreviations are used in this manuscript:

HRQoL	Health-Related Quality of Life
CRSwNP	Chronic Rhinosinusitis with Nasal Polyps
PROMs	Patient-Reported Outcome Measures
SNOT-22	22-item Sino-Nasal Outcome Test
NPS	Nasal Polyp Score
VAS	Visual Analog Scales
VIFs	Variance Inflation Factors
